# Supplementary material for: Variable microtubule architecture in the malaria parasite
Source: Nat Commun. 2023 Mar 3;14:1216. doi: 10.1038/s41467-023-36627-5 (PMC9984467; doi:10.1038/s41467-023-36627-5)
Supplement: Supplementary file 1 — Supplementary information [file 41467_2023_36627_MOESM1_ESM.pdf]

Supplemental Figures:

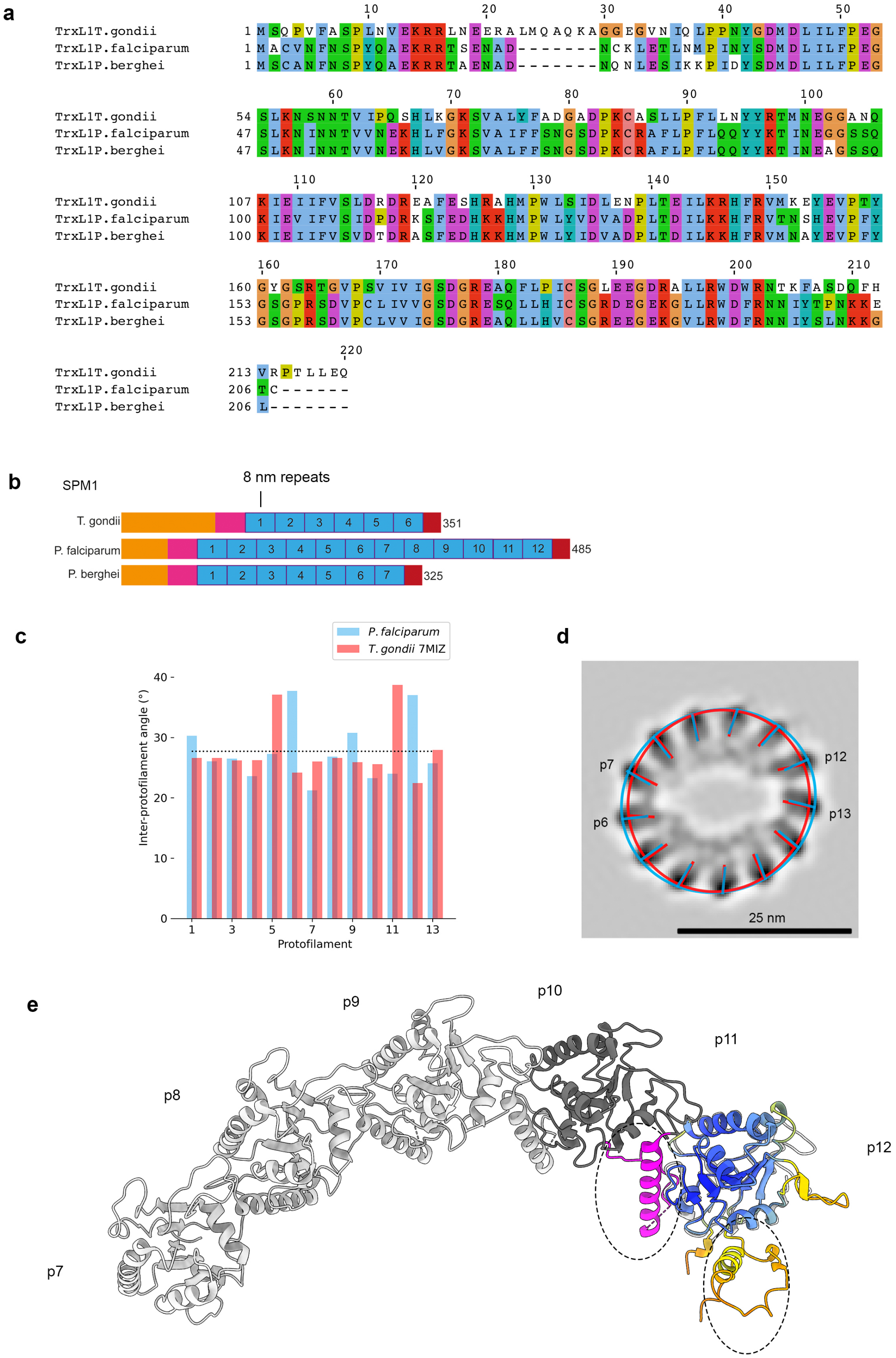

Fig. S1. The mosquito form microtubule is elliptical and has an interrupted luminal helix made up of the apicomplexan proteins TrxL1 and SPM1.
a. Multiple sequence alignment of TrxL1 protein from *T. gondii* (TGGT1_115220) and its homologues in *P. falciparum* (PF3D7_0919300) and *P. berghei* (PBANKA_0820200). Note the 7 amino acid insertion in *T. gondii*. **b.** Domain architecture of the SPM1 protein from *T. gondii* (TGGT1_263520) and its homologues in *P. falciparum* (PF3D7_0909500) and *P. berghei* (PBANKA_0810700) . SPM1 proteins are formed of a series of 32 amino acid repeats (roughly 8 nm long) which have been duplicated in *Plasmodium* from 6 in *T. gondii* to 7 in *P. berghei* and 12 in *P. falciparum.* **b** is adapted from Tran et al., 2012[1]. **c.** Angle relative to protofilament n+1. Dotted line denotes the inter-protofilament angle of a theoretical 13 protofilament microtubule. This was determined by fitting individual protofilaments of T. gondii tubulin into the *P. falciparum* density and measuring the angle relative to pdb 7MIZ. **d.** Both the *Toxoplasma gondii* (red) structure and our mosquito form *Plasmodium* (blue) structures have elliptical cross-sections. The average ellipticity (determined using the same measurements as in panel a) of a *Plasmodium* (blue ellipse) and *T. gondii* (red ellipse) microtubule and the relative tilt between protofilaments (blue and red bars) are superimposed onto a mean projection of the *Plasmodium* EM map. **e.** Predicted structure of PfTrxL1 (coloured based on prediction confidence) aligned to one subunit of *T. gondii* TgTrxL1, showing a good agreement. One TgTrxL1 subunit is coloured in dark grey, with the first N-terminal helix in magenta. The largest difference between the predicted PfTrxL1 and experimentally determined TgTrxL1 is at this N-terminal helix, which is responsible for a large part of the subunit-subunit interface. This helix is not visible in the experimental map at the gaps in the ILH (next to protofilaments 6 and 12), likely due to flexibility. Interestingly, there is a 7 amino acid insertion in the first helix in *T. gondii* (L23-A29) relative to *P. falciparum*, but most of these residues are not involved in forming the interunit interface.

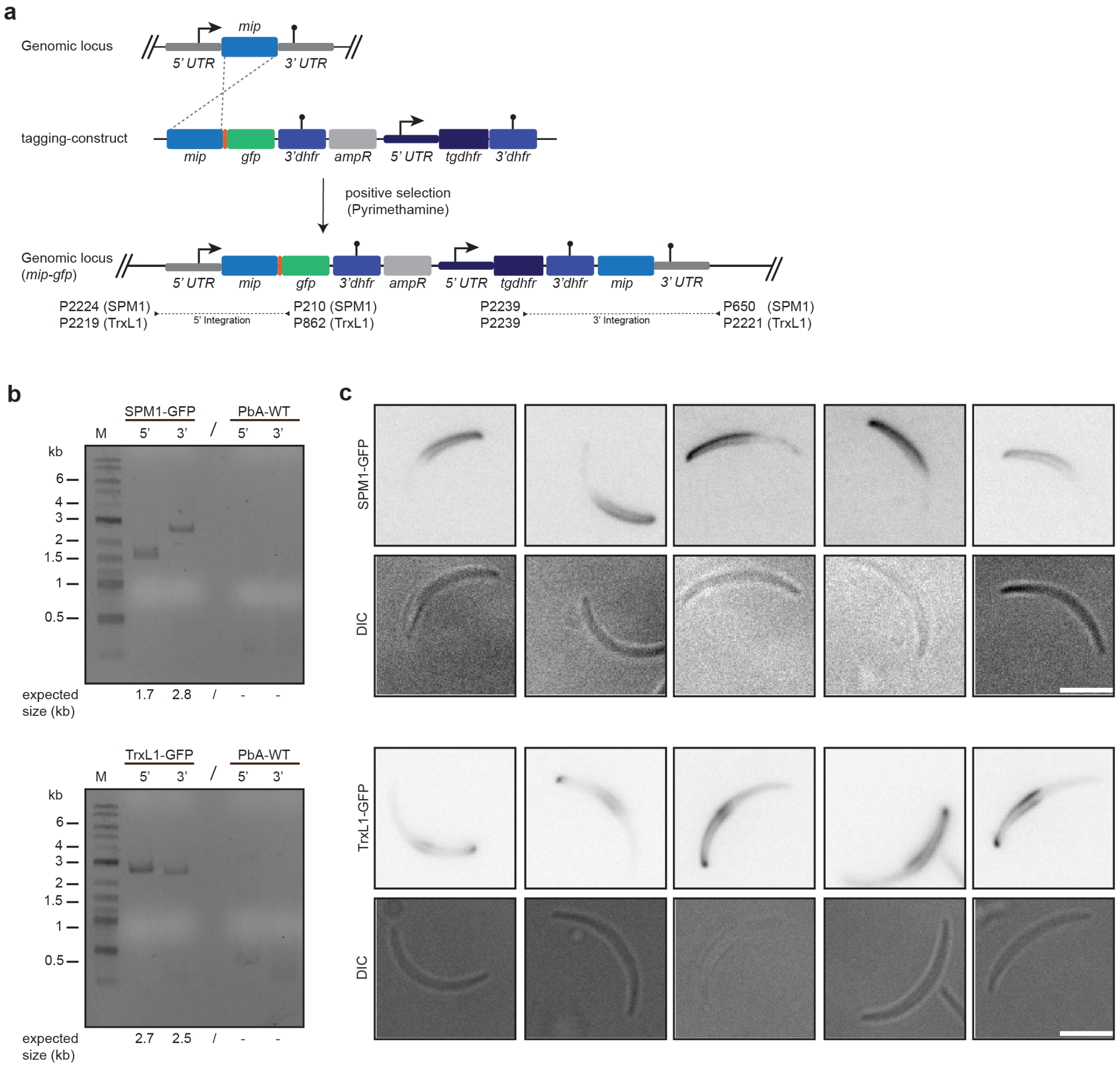

Fig. S2. Generation of *P. berghei* MIP-GFP parasite lines, validation and localization.
a. Schematic representation of the single-crossover strategy used to generate *Pb*SPM1-GFP and *Pb*TrxL1-GFP parasites. Primers used for genotyping PCR are indicated at the bottom (can be found in table S3). 3'dhfr = 3'UTR of dihydrofolate reductase; ampR = ampicillin resistance; gfp = green fluorescent protein; mip = microtubule inner protein; tgdhfr = *toxoplasma gondii* dihydrofolate reductase; UTR = untranslated region. **b.** Genotyping PCR to validate generation of *Pb*SPM1-GFP and *Pb*TrxL1-GFP in comparison to the parental *Pb*ANKA wildtype line. Primers used to verify 5' and 3' integration as indicated in A and expected amplicon sizes as indicated at the bottom of the gel images. **c.** Localisation of *Pb*SPM1-GFP and *Pb*TrxL1-GFP is consistent with SPMTs (peripheral localization for half to two-thirds of the cell length) as shown previously in Spreng et al[28]. Live cell imaging of salivary gland sporozoites dissected from 10-15 mosquitos for each line. Scale bars = 5 µm.

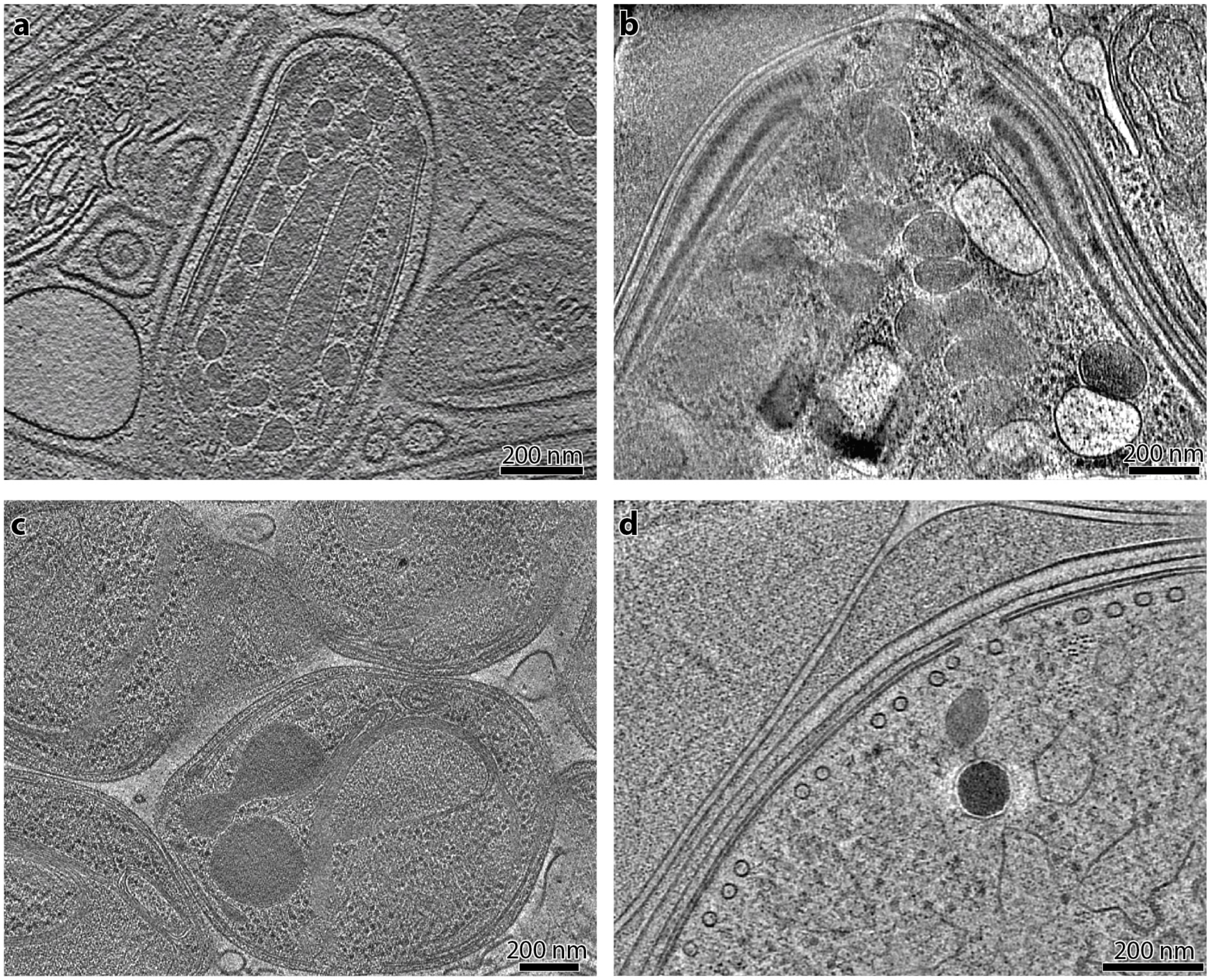

Fig. S3: Unannotated slices through tomograms that are segmented in the main figures. a. Sporozoite tomogram from Fig. 2f. **b.** Ookinete tomogram from Fig. 3e. **c.** Merozoite tomogram from Fig. 4c. **d.** Gametocyte tomogram from Fig. 5f.

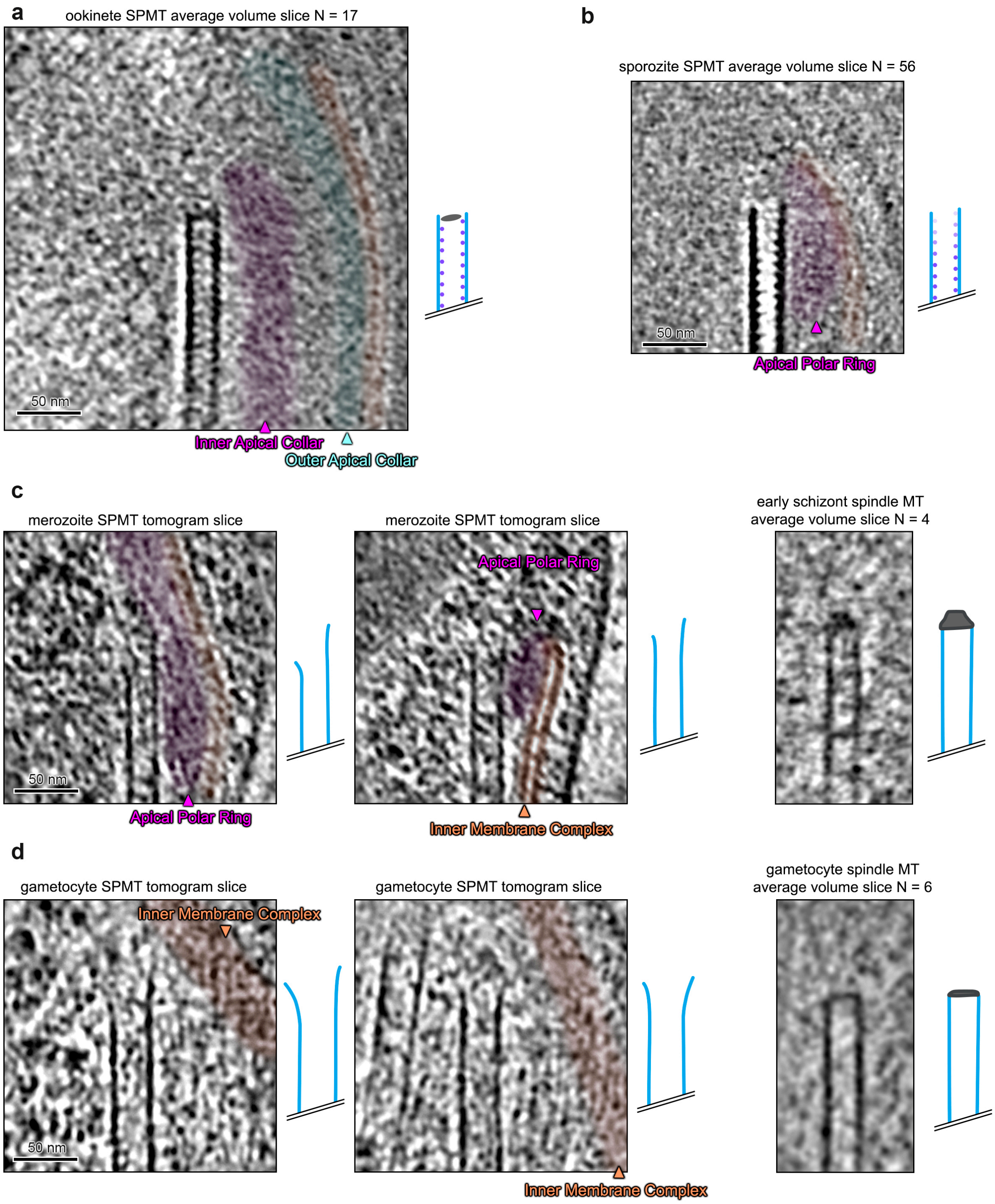

Fig. S4. Minus ends of SPMTs are uncapped, while nuclear spindle microtubules have a cap density consistent with γ-tubulin ring complex (γTuRC). The γ-tubulin ring complex (γTuRC) is a cone shaped complex consisting of 13 γ-tubulin subunits that presents binding sites to α- and β- tubulin. Cartoons to the right of greyscale images represent a model of the middle section through the respective microtubule minus ends. Number of particles included in SVA volumes are indicated above. IMC is highlighted in orange. **a.** Slice through an average volume of 17 minus ends of ookinete SPMTs. There was a hint of what could be a large protein complex

in the terminus lumen, but it could also be due to a SVA alignment artefact combined with a low particle number. **b.** Slice through an average volume of 56 minus ends of microtubules from sporozoites. The tapering intensity of ILH towards the terminus could be due to progressively lower TrxL1 occupancy. **c**. Left: Slice through two example subvolumes of merozoite SPMT minus ends. Right: A slice through an average volume of 4 minus ends of nuclear spindle microtubules from schizonts. **d.** Slices through two examples of minus ends of gametocyte SPMTs and an average volume of 6 minus ends of nuclear spindle microtubules from gametocytes. This volume is an ensemble average containing primarily 15 protofilament microtubules.

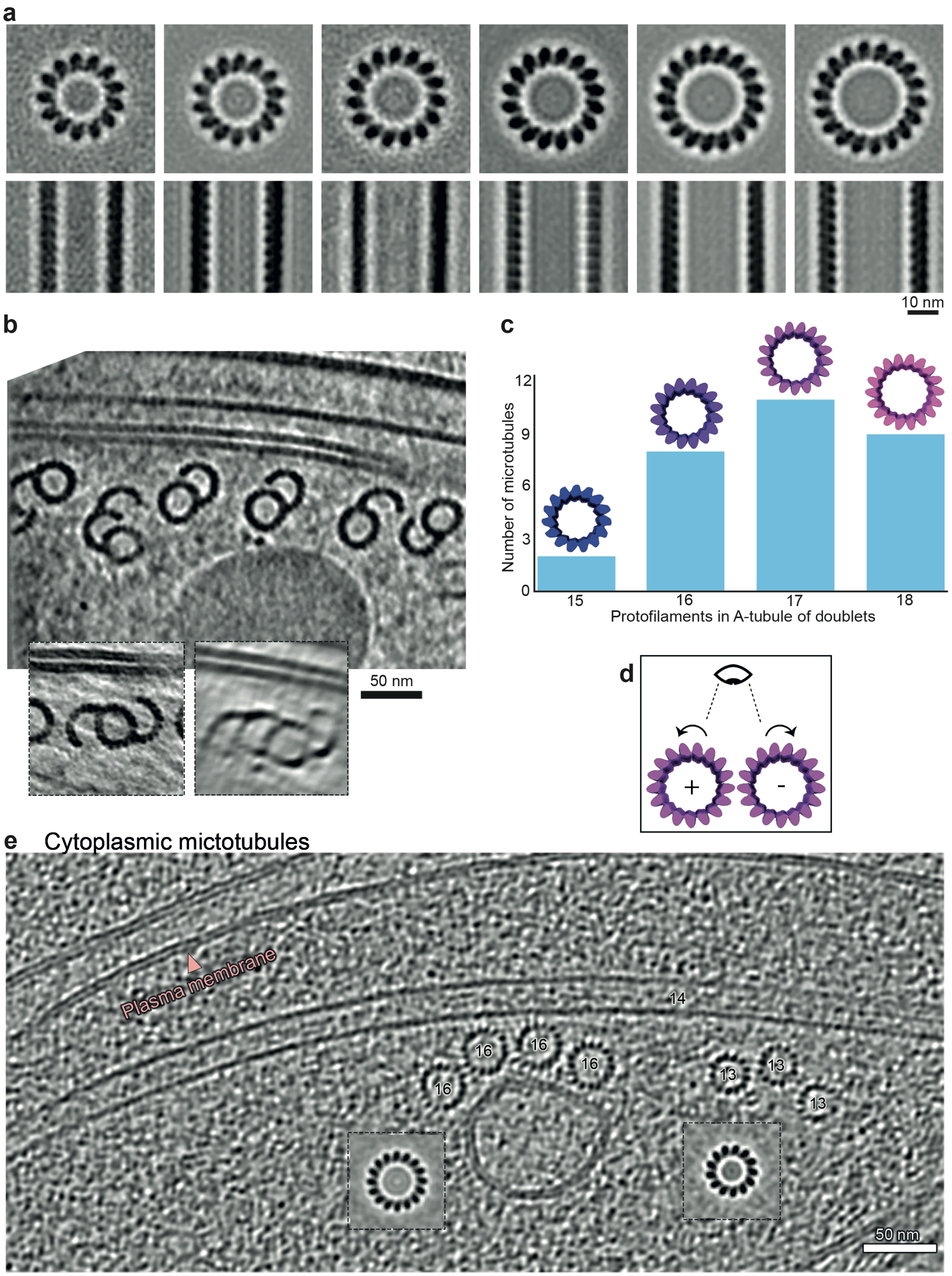

Fig. S5. Gametocyte SPMTs have random orientation and a range of protofilament numbers in singlets doublets, triplets and quadruplets. **a.** Slices through two orthogonal axes of EM maps of 13 to 18 protofilament SPMTs. **b.** Example slice through a subvolume showing doublets and triplets with different geometries and orientations relative to the nearby IMC. Inset: Example slice through a subvolume showing a triplet and quadruplet SPMT (observed smearing

is the result of missing tomographic information) **c.** Histogram of the protofilament number distribution in gametocyte singlets compared to doublet A tubules. **d.** Schematic showing how the polarity of a microtubule can be determined from the tilt direction of protofilaments. **e.** Slice through a subvolume of a stage III gametocyte cytoplasm showing cytoplasmic microtubules. In contrast to SPMTs, cytoplasmic microtubules appear clustered by protofilament number and polarity. Insets are examples of an average volumes of a single 13 protofilament microtubule and a single 16 protofilament microtubule from this tomogram.

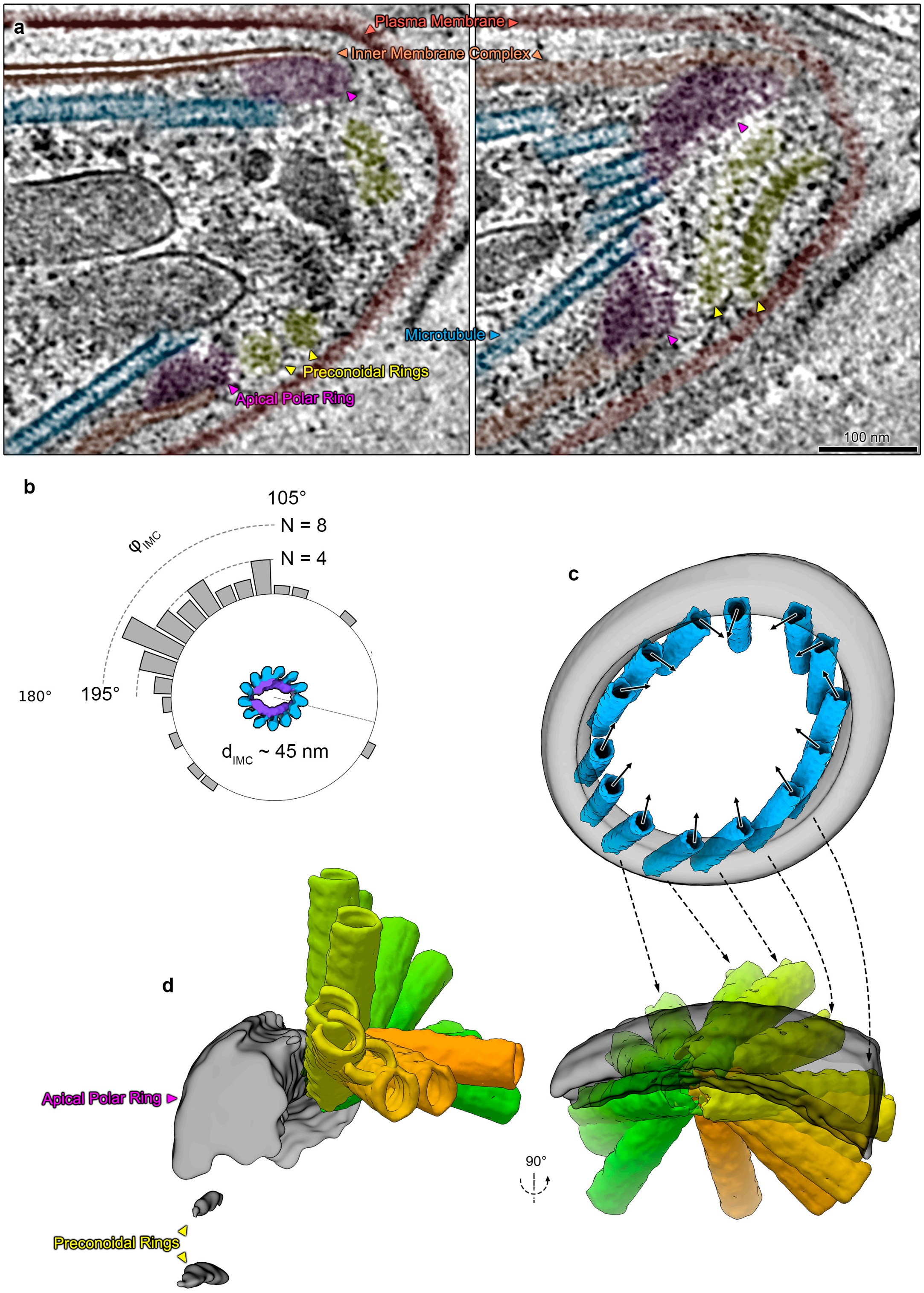

Fig. S6. Higher order organisation of subpellicular microtubules at the sporozoite apical pole. a. Two slices through the same tomogram of a sporozoite apex. Left: Slice through the centre of the APR and pre-conoidal rings. Right: slice through the edge of preconoidal rings and three SPMT minus ends. **b.** Histogram of individual SPMT radial orientations relative to the IMC (φIMC). As the closest point on the IMC could not be reliably determined due to the

toroidal shape of APR, φIMC was instead measured relative to the closest point on the APR determined by subvolume averaging. There is no significant difference between φIMC at apex and the cell body (main text Fig. 6B). **c.** Computationally smoothed SVA model of an APR (grey) with copies of map shown in B (blue) placed at coordinates determined by SVA. Vectors representing the orientation of the seam relative to SPMT centre are represented with arrows. Dashed arrows indicate corresponding SPMTs, not all are indicated. **d.** Superposition of SPMT minus ends (orange to green) showing their orientation relative to the closest segment of APR (grey) and illustrating the large degree of freedom in binding. This was generated by shifting and rotating each SPMT volume to the coordinate system of the nearest APR coordinate determined by SVA. Weak densities for preconoidal rings can be seen in the APR EM map, showing they are very roughly aligned with the APR.

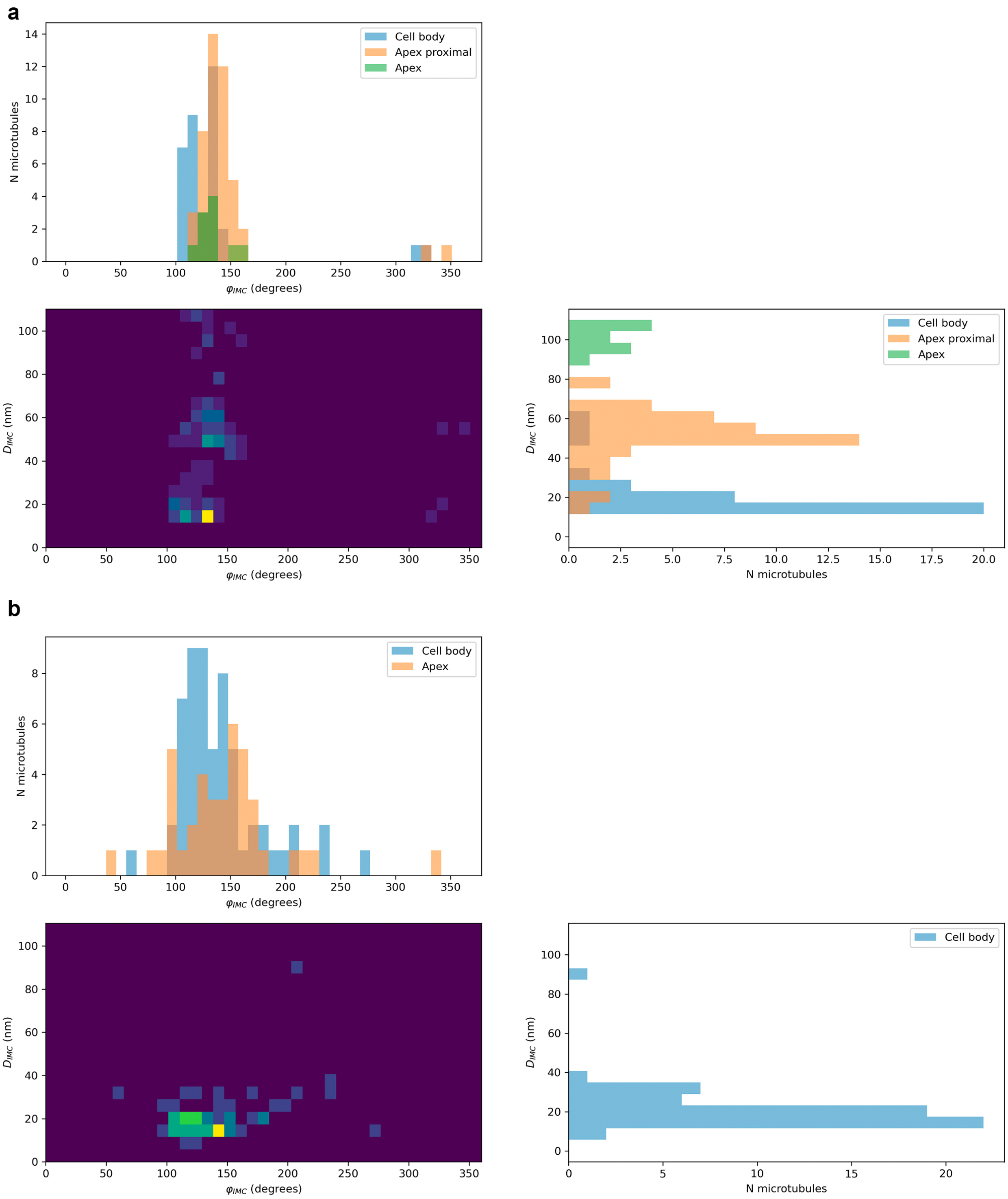

Figure S7: Mosquito form microtubules have a conserved distance and angle to the IMC. Full dataset from figure 6. Linear 2D and 1D histogram representation of seam-IMC angle (φ_{IMC}) and SPMT-IMC distance (d_{IMC}) also shown in Fig 6. **a.** Ookinetes. **b.** Sporozoites.

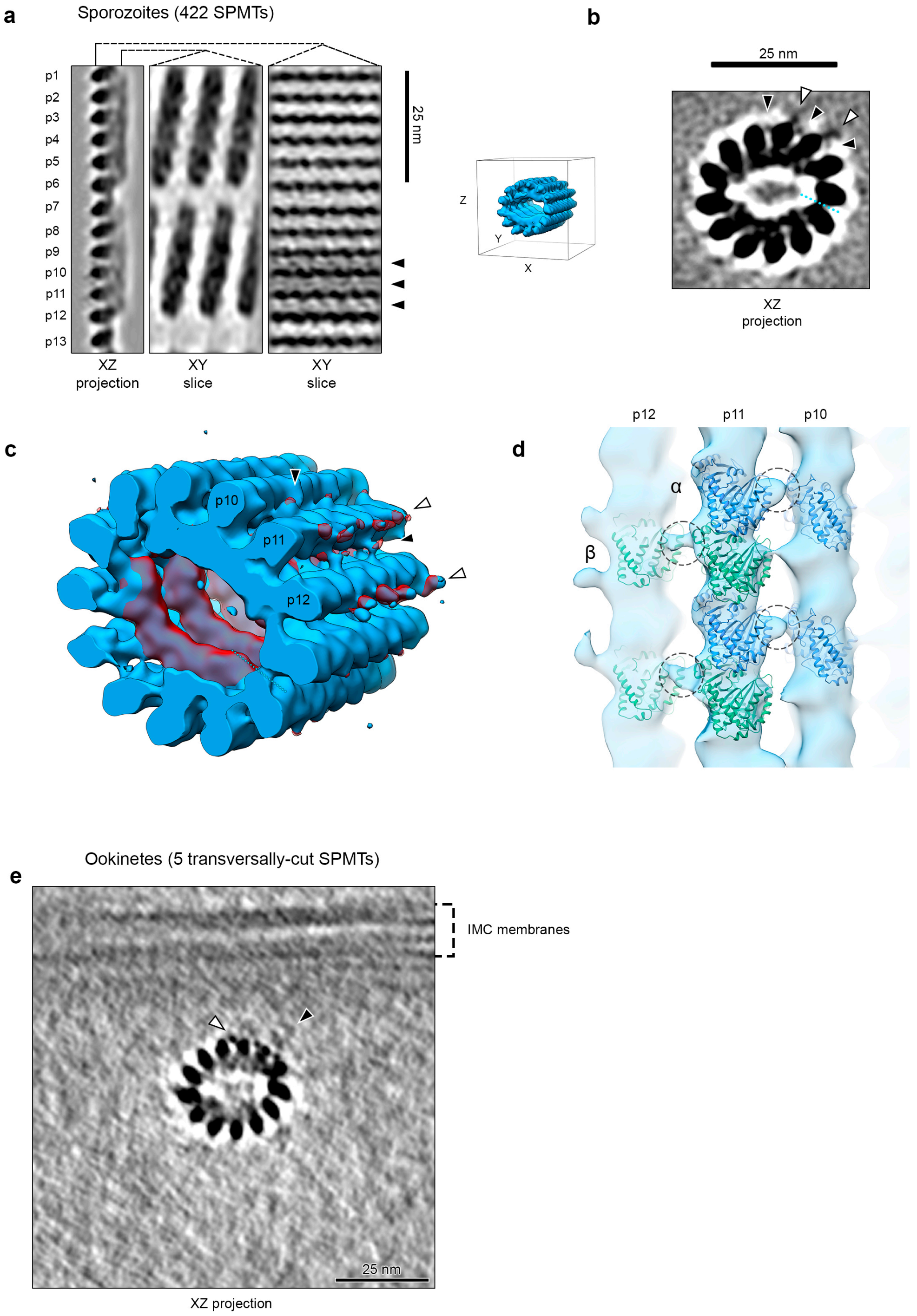

Figure S8. Mosquito form microtubules have a radially-asymmetric decoration with an unknown protein between protofilaments 10, 11 and 12. a. The sporozoite SPMT EM map was "unwrapped" (radially projected) and shown sliced along different axes. Left: XZ projection equivalent to the original map projection in B, protofilaments numbers are indicated (equivalent to numbering in Fig. 3C). Middle: Section through the ILH layer, equivalent to a "panoramic" view from the inside of the SPMT. Right: Section glancing the outermost part of protofilaments. Black arrowheads indicate densities between protofilaments (also in B). **b**. Left: EM map

isosurface with axes indicated to aid orientation. Right: Average projection through the EM map with contrast adjusted to highlight densities on protofilaments 10 and 11 (white arrowheads, also in C). **c**. Isosurface of SPMT EM map (blue) overlapped with a difference map (red) resulting from subtracting a simulated tubulin density map. **d.** Isosurface of three adjacent protofilaments with fitted tubulin model (7MIZ). Black dashed circles highlight extra densities between protofilaments. Note that these densities form a bridge between two α-tubulin subunits (between protofilaments 10 and 11) and two β-tubulin subunits (between protofilaments 11 and 12). **e**. Average projection through EM map generated by averaging 5 ookinete SPMTs that were cut at an almost perfect transversal orientation (i.e. their long axis was aligned with the electron beam at 0° stage tilt). The asymmetric densities seen in the sporozoite SPMT EM map (b) are clear, as well as the membranes of the IMC. This suggests that the densities emanating from protofilaments 11 and 12 are in fact parts of the SPMT-IMC link. However, most SPMTs are aligned with protofilaments 8 being closest to the IMC (i.e. the average orientation of the IMC is tilted ~ 45° to the right), indicating that there might be additional links to protofilaments 6 to 10 stabilising this orientation.

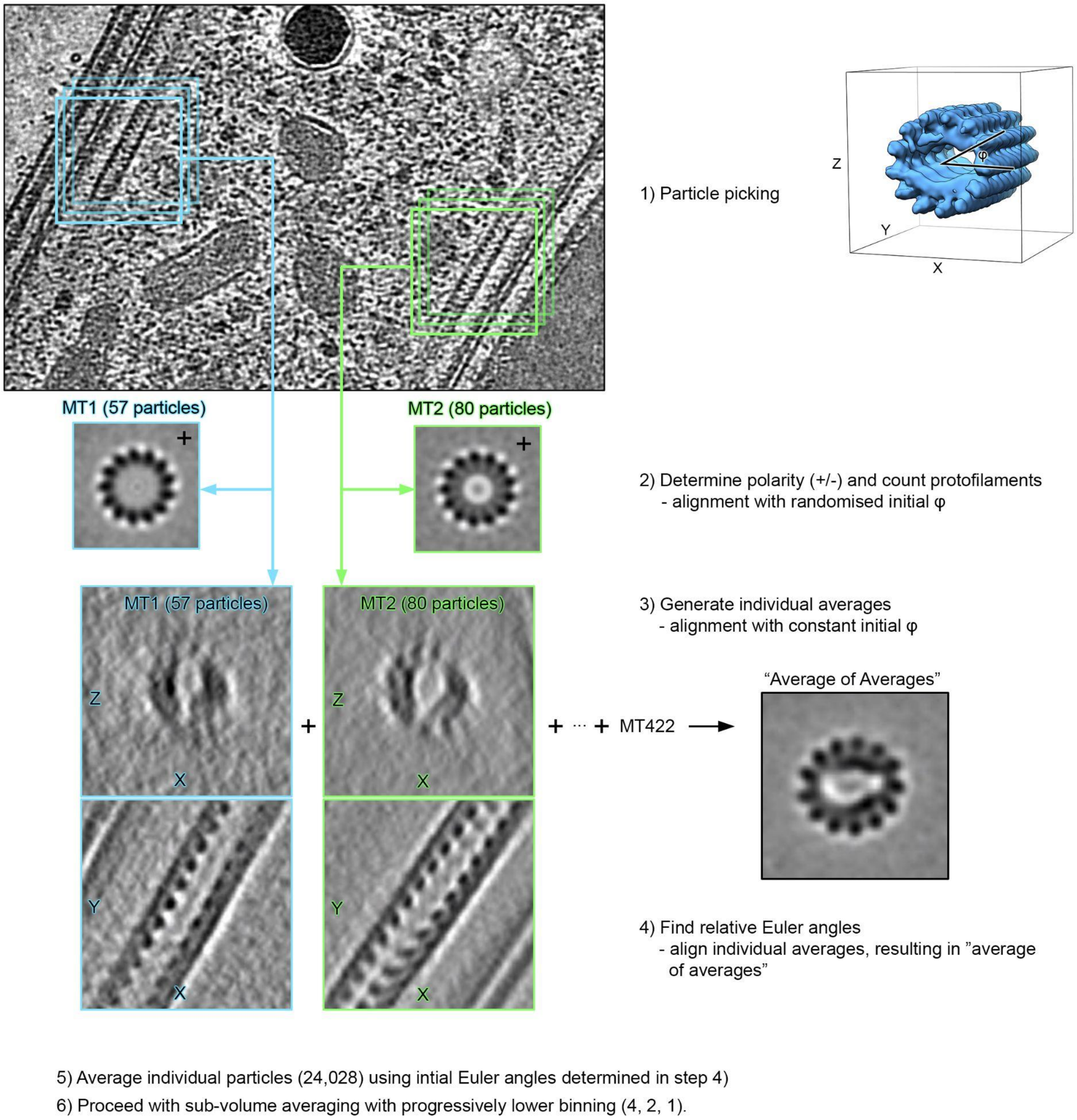

Figure S9: Schematic workflow for subvolume averaging of mosquito form SPMTs using sporozoite data as an example. 1) Microtubules were manually picked by tracing their centres, then interpolated to generate regularly spaced particles. 2) Each microtubule was processed individually with randomised initial φ angles (angle around pseudosymmetry axis) and these average volumes were used to determine the polarity of each microtubule (e.g. Fig. S5 D). 3) Each microtubule was averaged individually with constant initial φ angles, resulting in C1 average volumes. The missing tomographic information ("missing wedge") is apparent in these. 4) Individual average volumes from step 3) were now aligned together to make an "average of averages", thereby determining their relative φ rotation. 5) The newly determined φ angles were applied to individual particles. 6) SVA was performed at progressively lower binning until a final unbinned average was produced.

Supplementary tables.

Table S1.

tomo#	13pf -	13pf +	14pf -	14pf +	15pf -	15pf +	16pf -	16pf +	17pf -	17pf +	18pf -	18pf +
**1**	0	0	0	1	0	0	1	1	1	1	0	0
**2**	0	0	0	0	3	0	2	3	0	1	0	0
**3**	0	0	1	0	0	3	0	0	2	0	0	2
**4**	0	1	1	1	0	0	3	0	3	5	1	3
**5**	0	0	1	0	0	0	2	3	0	6	1	3
**6**	0	1	0	0	1	3	1	1	0	1	0	0
**7**	0	0	0	0	0	0	0	0	0	1	1	3
**8**	0	0	0	0	1	0	0	0	3	1	1	0
**9**	0	0	0	0	0	0	0	0	4	2	1	1
**10**	0	1	0	0	0	0	1	0	2	2	0	0
**11**	0	0	0	0	0	0	0	1	1	2	0	0
**12**	0	0	0	0	0	0	0	0	2	1	0	0
**13**	0	4	0	0	0	0	0	1	2	2	0	0
**14**	1	0	1	0	1	0	0	0	1	1	0	0
**15**	1	1	0	0	1	0	0	0	0	2	0	0
**16**	0	0	0	0	0	1	0	2	0	1	0	1
**17**	0	0	1	0	0	0	2	1	3	4	2	2
**18**	0	0	1	1	0	0	1	0	4	1	1	0
**19**	0	0	0	0	0	0	0	0	0	0	1	0
**20**	0	0	0	0	0	0	0	0	0	0	1	0
**21**	4	0	0	1	0	0	4	0	0	0	0	0

Polarities of gametocyte SPMTs in individual tomograms separated by protofilament number.

Table S2.

Parameter	Life cycle form			
	Sporozoites	Ookinetes	Gametocytes	Merozoites
Number of tomograms used for SVA	27	14	25	7
Number of individual microtubules in final average	407	159	Total: 152<br><br>13: 10<br>14: 10<br>15: 14<br>16: 31<br>17: 62<br>18: 25	Total: 12<br><br>Used in final average: 8
Total number of particles	13263	1851	13: 4053<br>14: 11806<br>15: 10435<br>16: 21459<br>17: 42738<br>18: 15045	8532
Pixel size (Å)	3.335	6.67 (2x binning)	6.67 (2x binning)	9.05 (2x binning)
Symmetry<br><br>Helical:<br>Pitch (degrees), helical rise (nm)	C1	C1	13: 27.7, 0.94<br>14: 25.77, 0.87<br>15: 24, 1.08<br>16: 22.5, 0.99<br>17: 21.17, 0.93<br>18: 20, 0.90	27.7, 0.94

Data collection parameters

Supplementary Text

Extended description of seam orientation in doublets for radial polarity measurement (Fig. 6)

We measured the angle φ_{IMC} between the seam, the microtubule centre and the closest point on the IMC surface. The first prerequisite for this relative angle measurement is the knowledge of the absolute position of the seam. This could be determined by SVA in ookinetes and sporozoites due to the asymmetric nature of the ILH. For the analysis of the radial polarity in gametocytes, we made use of the B tubules of doublets as a proxy for locating the seam. This relied on one of two

assumptions to be valid. The first is that the branch point is equivalent to that of canonical 13-protofilament doublets (i.e. between protofilaments n - 3 and n - 2, where n is the number of protofilaments in the A tubule[2,3]. Alternatively, the branch point could be in the same, but unknown, position relative to the seam regardless of the number of protofilaments in the A tubule. The latter assumption would invalidate the absolute values of measured angles but the relative distribution would still hold.

Supplemental references

1. Tran, J. Q., Li, C., Chyan, A., Chung, L. & Morrissette, N. S. SPM1 Stabilizes Subpellicular Microtubules in Toxoplasma gondii. *Eukaryot. Cell* **11**, 206–216 (2012).
2. Li, S., Fernandez, J.-J., Fabritius, A. S., Agard, D. A. & Winey, M. Electron cryo-tomography structure of axonemal doublet microtubule from Tetrahymena thermophila. *Life Sci. Alliance* **5**, e202101225 (2021).
3. Ma, M. *et al.* Structure of the Decorated Ciliary Doublet Microtubule. *Cell* **179**, 909-922.e12 (2019).
