# Supplementary material for: Variable microtubule architecture in the malaria parasite
Source: Nat Commun. 2023 Mar 3;14:1216. doi: 10.1038/s41467-023-36627-5 (PMC9984467; doi:10.1038/s41467-023-36627-5)
Supplement: Supplementary file 3 — Description of additional supplementary files [file 41467_2023_36627_MOESM3_ESM.docx]

**Description of additional supplementary files**

**Title: Movie S1** **Sporozoite tomogram.**

Description: Movie slicing through a tomogram of a sporozoite. Volume shown in Fig. 2f.

**Title: Movie S2 Ookinete tomogram.**

Description: Movie slicing through a tomogram of an ookinete. Volume shown in Fig. 3e.

**Title: Movie S3 Schizont tomogram.**

Description: Movie slicing through a tomogram of a schizont. Volume shown in Fig. 4c.

**Title: Movie S4 Gametocyte tomogram.**

Description: Movie slicing through a tomogram of a gametocyte. Volume shown in Fig. 5f.
